# Supplementary material for: MetaRibo-Seq measures translation in microbiomes
Source: Nat Commun. 2020 Jun 29;11:3268. doi: 10.1038/s41467-020-17081-z (PMC7324362; doi:10.1038/s41467-020-17081-z)
Supplement: Supplementary file 10 — Supplementary Data 7 [file 41467_2020_17081_MOESM10_ESM.zip › File2/Confidence_High_Taxonomy/340450_out.krona.html]

Javascript must be enabled to view this page.

members
magnitude
magnitudeUnassigned
count
unassigned
taxon
rank

340450\_out

1

SRS065310\_contig\_number\_contig-100\_12934.12934
2

superkingdom
2
1

phylum
1239
1

class
91061
1

order
1
186826

family
1
1300

1
1301
genus

species group
671232
1


SRS063215\_contig\_number\_7019
1
1328
species
